# Summary of the proceedings of the International Forum 2021: “A more visible radiologist can never be replaced by AI”

**DOI:** 10.1186/s13244-022-01182-y

**Published:** 2022-03-14

**Authors:** European Society of Radiology (ESR), M. C. Mahoney, G. McGinty, G. M. Figueroa Sanchez, N. Rodríguez Pedraza, M. Arrieta Usta, V. Muglia, M. Brandão da Costa, B. E. González Ulloa, T. El-Diasty, Usama AlBastaki, C. Amarnath, W. Tanomkiat, J. Chaiyakum, S. Liu, S. H. Park, S. Aoki, D. Varma, L. Lawler, A. Rockall, R. A. Mendonça

**Affiliations:** European Society of Radiology, Am Gestade 1, 1010 Vienna, Austria

**Keywords:** Artificial intelligence, Visibility, Technology, Future

## Abstract

The ESR International Forum at the ECR 2021 discussed effects of artificial intelligence on the future of radiology and the need for increased visibility of radiologists. The participating societies were invited to submit written reports detailing the current situation in their country or region. The European Society of Radiology (ESR) established the ESR International Forum in order to discuss hot topics in the profession of radiology with non-European radiological partner societies. At the ESR International Forum 2021, different strategies, initiatives and ideas were presented with regard to radiology community’s response to the changes caused by the emerging AI technology.

## Key points

- AI and radiology should be understood as having a symbiotic rather than a competitive relationship.
- The use of AI can increase the visibility of radiologists and allow them to focus on other issues and expand their current role.
- Use of AI can significantly increase the level of care offered to patients.
- Radiologists need to be viewed as thought leaders and medical experts in AI.
- The advent of AI can propel radiology into a high visibility technology-enabled speciality.

## Introduction

The ESR International Forum was established by the European Society of Radiology with the aim to discuss pivotal themes in the field of radiology with international societies from outside Europe. The ESR International Forum is held every year at the European Congress of Radiology (ECR), and participation is by invitation only. Due to the global pandemic of COVID-19, ECR 2021 was again held as a virtual congress and the participants of the ESR International Forum submitted written reports describing their society’s approach to the important issue of the use of AI in radiology.

Previous topics discussed in the ESR International Forum include measures undertaken by radiologist in the fight against COVID-19, the relation between radiology and nuclear medicine, the position of ultrasound in radiology, the relation of general radiology and subspecialty radiology, the implementation of clinical decision support and imaging referral guidelines in the clinical routine, the position of interventional radiology within radiology, value-based radiology, and the strategies to engage the younger generation.

The following societies submitted a report and presented the point of view of their respective country or region: The American College of Radiology (ACR), the Asian Oceanian Society of Radiology (AOSR), the Brazilian College of Radiology (CBR), the Chinese Society of Radiology (CSR), the Colombian Association of Radiology (ACR), the Egyptian Society of Radiology and Nuclear Medicine (ESRNM), the Indian Radiological and Imaging Association (IRIA), the Inter-American College of Radiology (CIR), the International Society of Radiology (ISR), the Japan Radiological Society (JRS), the Korean Society of Radiology (KSR), the Mexican Federation of Radiology and Imaging (FMRI), the Mexican Society of Radiology and Imaging (SMRI), the Radiological Society of the Emirates (RSE), the Radiological Society of North America (RSNA), the Radiological Society of Saudi Arabia (RSSA), the Radiological Society of Thailand (RST), the Royal Australian and New Zealand College of Radiologists (RANZCR), the Royal College of Radiologists of Thailand (RCRT), and the Radiological and Diagnostic Imaging Society of São Paulo (SPR). The European Society of Radiology (ESR) presented the situation in Europe.

## The situation in North America

M. C. Mahoney submitted the report on behalf of the Radiological Society of North America (RSNA). Pointing out that the RSNA recognized early on the importance of harnessing the power of AI to advance radiology, improve workflow and enhance patient care, M. C. Mahoney explained that the RSNA leads the AI charge, convening radiologists, data scientists and industry toward the shared goal of gathering, organizing and labelling volumes of data to help solve some of today’s most pressing health problems. Core RSNA AI-related activities are done through the RSNA annual meeting, peer-reviewed journals and data-gathering initiatives and collaborations. In 2020, RSNA’s first all-virtual meeting offered more than 100 AI education and research sessions. M. C. Mahoney further emphasized that the RSNA’s online journal *Radiology: Artificial Intelligence*, launched in 2019, highlights emerging applications of machine learning and artificial intelligence in the field of imaging across multiple disciplines and keeps practicing physicians and imaging researchers up to date on the best emerging science in this subspecialty. Additionally, RSNA’s flagship journal *Radiology* also publishes cutting-edge AI research. In mid-2020, the RSNA COVID-19 AI Task Force launched the RSNA International COVID-19 Open Radiology Database (RICORD), envisioned as the largest open database of anonymised COVID-19 medical images in the world and in December 2020, the first annotated dataset from RICORD was published by The Cancer Imaging Archive (TCIA). M. C. Mahoney also explained that each year since 2017, RSNA has organized “AI Challenges” to spur the creation of AI tools for radiology. Finally, M. C. Mahoney concluded that radiologists’ participation in and contributions to patients’ healthcare teams are vital and that AI has enormous potential to make radiologists even more visible and valued members of those healthcare teams.

G. McGinty, on behalf of the American College of Radiology (ACR), emphasized that radiologists, as both experts in imaging and leaders in the application of data science to health care, are the best equipped physicians to lead healthcare teams augmented by machine learning. Pointing out that in the USA there continues to be continued high demand for radiologists [1], G. McGinty explained that it has become clear that radiologists are critical to developing and implementing AI in ways that will be safe, efficient and effective for the patients. Even though the US Food and Drug Administration (FDA) has cleared over 100 AI algorithms for clinical use in imaging, they all require final interpretations by physicians. G. McGinty stated that autonomously functioning AI will not occur until machines are also able to analyse and synthesize clinical history and inputs from all relevant data sources and that radiologists will best preserve their unique role by ensuring that they are present as trusted stewards of technology throughout the process of care from determining appropriateness of imaging examinations to effectively communicating results. Commenting on the ACR AI-related initiatives, G. McGinty explained that the American College of Radiology's Imaging 3.0 initiative [2] and its Commission on Patient and Family Centered Care [3] provide radiologists examples of increasing their visibility and value to patients and health systems. Despite the advances in AI, the general population does not yet support independent use of AI for image interpretation [4] and radiologists will need to serve as a visible resource to patients as AI tools assume more importance in diagnosis to ensure that patients gain access to the highest quality care. The ACR has also collaborated with other professional societies on a road map for the ethical use of AI in imaging [5] and includes the voices of patients through its committees and advisory groups. Finally, G. McGinty emphasized that radiologists need to be viewed as thought leaders and medical experts in AI. In that sense, the ACR’s Data Science Institute (DSI) [6], founded in 2017, provides education as well as tools to evaluate and monitor algorithms, empowering radiologists to assume leadership roles in delivering ethical, patient-centred, AI-enabled care focused on equity, quality and outcomes.

## The situation in Latin America

G. M. Figueroa Sanchez, on behalf of the Mexican Federation of Radiology and Imaging (FMRI), reported on the current situation in Mexico. He pointed out that radiologists have a unique opportunity to become more visible thanks to artificial intelligence. By already managing various different systems, such as Radiology Information System (RIS) and Picture Archive and Communication System (PACS), and considering teleradiology, interdepartmental sessions, tumour boards, teleconsultations, etc., radiologists have the possibility to drive the technological revolution. Even though many processes are probably going to be handled by AI in the future, G. M. Figueroa Sanchez emphasized that radiologists would have a crucial role in the complex process of integrating all information, putting it in the context of the patient and offering meaningful interpretation for the appropriate diagnosis and treatment. G. M. Figueroa Sanchez particularly emphasized that a visible radiologist adds value in the interpersonal relationship with patients, their families and also with clinical colleagues. He further stated that radiologists are to become data scientists, playing a key role in Precision Medicine, implementing the applications of AI and Big Data, thus being a fundamental part of the health system. Finally, G. M. Figueroa Sanchez stated that an AI-empowered radiologists will better communicate diagnosis to patients and referring physicians, will be more creative, empathic, with intuition and imagination and that being visible in the near future will certainly entail becoming experts in teleradiology and developing web-based portals with and for patients.

N. Rodríguez Pedraza on behalf of the Mexican Society of Radiology and Imaging (SMRI) stated that radiology as a specialty has experienced an unprecedented progress; however, that recent advances in technology have made the radiologist more vulnerable, which is best reflected in the lack of communication between radiologists and patients and referring physicians. N. Rodríguez Pedraza emphasized that while the processing of data and medical images through the AI algorithms has helped in reducing the burden of repetitive tasks and has increased overall diagnostic accuracy, the absence of contact between radiologists and patients still remains an issue. She further pointed out that the SMRI implemented AI modules in its congress in order to ensure that Mexican radiologists are more acquainted with the automation of data and can in turn improve diagnoses without losing the human perspective. Furthermore, the SMRI is developing multidisciplinary webinars and it is intended to apply this multidisciplinary approach to future congresses as well, which will in turn make radiologists more visible by putting them in contact with various other specialties involved in the diagnosis of a condition.

Finally, N. Rodríguez Pedraza stated that including the name and a photograph of the physician interpreting the image on the X-ray film would make them more visible.

M. Arrieta Usta, reporting on behalf of the Colombian Association of Radiology (ACR), stated that currently many patients are not aware of the usefulness of diagnostic imaging studies and imaging procedures, with some of them also not knowing the role of the radiologist and other professionals in the treatment and diagnosis of their medical conditions. Additionally, many other factors, such as isolation, anonymity, high workflows in short time, diagnosis by other physician and improper administrative protocols, affect radiologists. And, with the application of artificial intelligence, radiologists are at risk of becoming even more distant from the patient. That is why M. Arrieta Usta pointed out the importance of person-centred care. In that sense, the ACR has created “Radiology for life”, a community of patients that allows ACR to inform patients about various topics related to diagnosis and image intervention. M. Arrieta Usta explained that through this community, the ACR is developing a series of actions that allow radiologists to approach patients, highlight the importance of radiology, diagnostic images and therapy by images (interventional radiology), in addition to highlighting the role of professionals linked to the specialty. The ACR has also developed various scientific groups, one of which is Artificial Intelligence scientific group, coordinated by a committee consisting of an interdisciplinary team that works toward the proper use of artificial intelligence tools in the health sector in Colombia, in addition to promoting and supporting the developments in this area. Finally, M. Arrieta Usta explained that since 2019 the ACR has been venturing into the field of artificial intelligence through the promotion of different academic fora, in which issues related to the impact of these technologies on the exercise of radiology and diagnostic images could be discussed. In addition to education, the ACR Artificial Intelligence committee has focused its activities on three specific thematic pillars: regulation, ethics and technical aspects.

V. Muglia, on behalf of the Brazilian College of Radiology (CBR), reported on the situation in Brazil. He explained that as diagnostic imaging is intrinsically linked to the development of new technologies its transformation through technological advances is inevitable. This is in turn also reflected in communication with other specialties and patients. V. Muglia stated that modern radiologists should increase their visibility by being present in multidisciplinary rounds, by being always available for consulting complex cases and by being accessible for patients. Pointing out that patients’ expectations are essential for changing how radiologists are seen, V. Muglia emphasized that radiologists should be readily available and should provide a good experience for patients in radiology centres, while also providing rapid turnover of reports. He further explained that the idea of AI as more of an ally than a threat to radiology is slowly gaining track. After an initial period in which AI was viewed as the panacea for solving all the problems of medicine, currently the irreplaceable role of the radiologist as the professional who is able to provide the best interpretation for imaging findings is being less questioned. V. Muglia also stated that currently several challenges prevent AI’s use on larger scale, namely the issue of generalisability and the model bias. However, it is possible to identify AI tools that can perform the simplest and most repetitive tasks of searching imaging findings, allowing radiologists to focus on the most complex role of finding the best interpretation. Commenting on the role of radiological societies, V. Muglia explained that they need to lead the narrative of the fundamental role of Diagnostic Imaging in patient’s care and emphasize the importance of Value-Based Health Care. Finally, V. Muglia stated that the notion that radiologists have a central role in clinical care and would not be replaced by AI should be strongly and repetitively delivered.

M. Brandão da Costa, reporting on behalf of the Radiological and Diagnostic Imaging Society of Sao Paulo (SPR), pointed out that the use of AI allows for greater efficiency in regard to diagnostic imaging and helps radiologists find a more precise diagnose. In that sense AI will never replace radiologists but will allow them to become better professional who help patients more efficiently.

B. E. González Ulloa, representing the Inter-American College of Radiology (CIR), explained that radiologists, who were on the forefront of the digital era in medicine, can guide the introduction of AI into health care. They will, however, not be replaced because radiology includes communication of diagnosis, consideration of patient’s values and preferences, medical judgment, quality assurance, education, policy making and interventional procedures. The higher efficiency provided by AI will allow radiologists to perform more value-added tasks, becoming more visible to patients and playing a vital role in multidisciplinary clinical teams. Commenting on the creation of algorithms used in radiology, B. E. González Ulloa stated that scientists creating them might need to learn more about medicine which is something where radiology professionals could serve as guides. Proper understanding of the limitations of algorithms by clinicians and proper understanding of clinical data by programmers is key to creating algorithms usable in the clinical work. In conclusion, B. E. González Ulloa stated that in Latin America, like other regions of the world, clinical radiologists play an important role in diagnosis integration. AI and its applications on imaging are powerful tools designed to increase diagnosis accuracy but will not replace radiologists.

## The situation in Egypt

T. El-Diasty, on behalf of the Egyptian Society of Radiology and Nuclear Medicine (ESRNM), stated that it is important to explore the role of the radiologist in building AI models in collaboration with the engineering teams. In that sense the current role of the radiologists could be additionally expanded as radiologists can provide additional valuable information to engineers. Furthermore, T. El-Diasty mentioned an example of the Urology and Nephrology Centre at the Mansoura University, Egypt, that has an outstanding collaboration with an engineering team from the University of Louisville, KY, USA. This collaboration was established in 2004 and is still ongoing in many different projects related to the early detection and diagnosis of different diseases. T. El-Diasty concluded his report by stating that AI is there to assist radiologists and the radiologists and AI will continue to complement each other in the future.

## The situation in Saudi Arabia

L. Jamjoom, on behalf of the Radiological Society of Saudi Arabia (RSSA), explained that it is important for a visible consultant to be accessible, affable and accomplished that a visible radiologist that uses AI will never be replaced by it. L. Jamjoom emphasized that AI has a complementary role and helps with tedious points in workflow, resulting in improved patient outcome, and enables radiologists to spend more time on what is important. Furthermore, she mentioned that a survey on AI, conducted in Saudi Arabia, examined radiologists’ attitude towards AI and that many radiologists found that AI will increase overall efficiency by reducing analysis and reporting time, enhance the diagnostic accuracy and provide medical facilities to remote areas, help with triaging and pinpointing alarming findings, improve health care, decrease diagnostic errors and will increase standardization. At the same time radiologists were worried that AI might cause a reduction in the number of doctors and specialists, increase the number of false results and lead to stereotyping and stiffness, wrong pattern recognition, decrease in the need for primary care physicians, lack of accountability and decrease when it comes to the communication with patients and case discussion. Finally, L. Jamjoom stated that Saudi Arabian expenditure on AI projects accounts for 63% of global investments and that AI implementation will involve a total of 14 hospitals and 56 small healthcare centres.

## The situation in the United Arab Emirates

Dr. AlBastaki, on behalf of the Radiology Society of the Emirates (RSE), explained that it is important to note that with the increase in the use of AI radiologists should have a more effective role in supervising the results procured by the AI. In that sense, radiologists should not feel threatened by AI but should instead consider it as a chance to evolve and focus on other issues. Dr. AlBastaki pointed out that radiologists should be receptive to the new developments and should offer continuous feedback for the developers to improve the systems. Finally, Dr. AlBastaki mentioned that currently in the United Arab Emirates there are more than 5000 chest X-Rays performed daily, which leads to great pressure on radiologists in the country and is something where future AI developments will certainly help.

## The situation in India

C. Amarnath, on behalf of the Indian Radiological and Imaging Association (IRIA), emphasized that radiologists have always adopted and adapted to major technological changes, digitalisation and new modalities. He pointed out that one of the main roles of the radiologist is to serve as a bridge between clinicians and patient diagnosis. However, with clinicians’ dependence on imaging increasing exponentially, studies have become more complex, and at the same time, there is a huge surge in volume, as witnessed during the current COVID-19 pandemic. Expectations towards radiologists have escalated with need of high-resolution data, 3D reconstructions, specific reporting standards, quantitative parameters, frequent follow-ups, quality control and research. Pointing out that imaging has become a commodity in medical practice, C. Amarnath stated that radiologists need to emerge as the conductors of multi-disciplinary teams at the forefront of clinical care and that this is something where AI, as an *intelligent assistant* to radiologists, could help. C. Amarnath explained that in this role AI could bring efficient synergies to radiology: reduce radiologists’ visual fatigue, address the exponential workload burst, triage normal, delegate repetitive tasks to machines and accomplish tasks that do not need human intervention. Reporting on the situation in India, C. Amarnath reported that the AI-division of IRIA is aiming to identify the needs and demands in India, generate new ideas and use clinical data to accomplish algorithms and build products. He also stressed the importance of introducing some sort of governance for decision-making based on autonomous AI-based algorithms. New policies regarding regulation of data protection, privacy of sensitive information, cybersecurity, accountability and responsibility issues need to be addressed as well. C. Amarnath concluded his report by stating that it is high time for radiologists to reclaim lost ground, utilize AI to optimize workflow, re-establish their role in clinical interdisciplinary teams for value added work, decision making and intervention.

## The situation in Thailand

W. Tanomkiat, reporting on behalf of the Royal College of Radiologists of Thailand (RCRT), stated that RCRT established a dedicated committee, consisting of radiologists, data scientists and data analysts, in order to study the impact of AI on diagnostic radiology, advise and recommend best courses of action to the RCRT Broad of Directors. The Committee quickly recognized that AI will inevitably affect radiology in Thailand and the RCRT announced the first version of the suggestions for AI users in diagnostic radiology and signed a Memorandum of Understanding (MOU) on exchanging the resources with the Thailand Centre of Excellence in Life Sciences (TCELS) [7]. W. Tanomkiat explained that early introduction of AI to members of the RCRT has been done through the “RadioVolunteer”, a non-profit project organized by the RCRT, TCELS, J.F. Advance Med Company and Department of Corrections [8]. The project focused on the analysis of chest radiographs of the prisoners infected with COVID-19 in Thailand. With more than 1000 chest radiographs per day, radiologists in certain communities were feeling overwhelmed, so a digital platform was developed where radiologists from the entire country could log in and offer their expertise, aided by AI if requested. W. Tanomkiat further explained that a structured report form was specially designed to direct prioritization of the patients and was digitized to speed up the process of making the statement, leading to an output of more than 1500 chest radiographs a day or over 30 chest radiographs per minute. W. Tanomkiat concluded his report by stating that the next steps of the RCRT will include adjusting the education curriculum and residency training.

J. Chaiyakum, on behalf of the Radiological Society of Thailand (RST), reported that the RST sent a questionnaire to Thai radiologists, to survey their opinions regarding the effect of AI on their practice and income. Almost 80% of the respondents stated that AI currently has no role in their workplace, and out of the remaining 20% most respondents stated that AI is used mostly for screening and as a help with detecting lesions. It was reported that more than 2/3 of respondents believe that AI will play a significant role in the future, in particular when it comes to the aforementioned issues of screening and detecting lesions. Radiologists in Thailand are overall not fully concerned that they will be replaced by AI and more than 50% believe that their income will not be decreased or will be only slightly decrease due to AI. Finally, J. Chaiyakum reported that a majority of radiologists would encourage young doctors to pursue a career in radiology.

## The situation in China

S. Liu, on behalf of the Chinese Society of Radiology (CSR), reported on the situation in China. He began his report by stating that the basic consensus that radiologists and AI are in a symbiotic rather than a competitive relationship has long been existing in China. Therefore, a focus was placed on how AI can assist doctors in China and as a result number of radiologists using AI as an assisting tool is increasing year by year. Quoting Dr. Curtis Langlotz, a radiologist from Stanford who stated that “AI won’t replace radiologists, but radiologists who use AI will replace radiologists who don’t”, S. Liu explained that this is becoming reality in China. Commenting on the notion of potential *replacement* of radiologists by the AI, S. Liu emphasized that radiologists are becoming more dependent on AI in some specific scenarios after few years of applications, but also have to dedicate more time to reassuring the patients and collaborating with clinicians. Thus, AI is not replacing radiologists’ “traditional work” but is extending their work boundaries. Finally, S. Liu concluded that AI has brought a profound change to the industry of medical imaging and that those who accept change, embrace change, and accommodate to change, are the ones who will own the future.

## The situation in Korea

S. H. Park submitted a report on behalf of the Korean Society of Radiology (KSR). He explained that main activities of the KSR regarding AI include educational programs, not only for radiologists but also for industry and government agencies, as well as making policy suggestions by collaborating with government agencies regarding the regulatory approval, insurance coverage and clinical implementation of AI devices. One of the central positions that KSR has in those AI-related activities is "human in the loop". As the strengths of humans and AI are different, it is critical both technically and ethically to include them both in order to ultimately improve health care. S. H. Park stated that Korean radiologists are overall no longer swayed by the initial hype regarding their replacement by AI and are instead examining how they can engage with and embrace AI to improve radiology practice for the good of the patients. Consequently, they understand that any substitution of radiologists by AI only occurs if radiologists cannot be found when needed which is why visibility is of utmost importance. S. H. Park also reported that in 2020 the KSR accomplished several AI-related projects, including an e-learning program on AI as well as a mini-textbook on AI in health care. S. H. Park concluded his report by stating that the KSR plans to expand its scope of AI education by designing a more in-depth, hands-on course that aims to train radiologists who could serve as leaders for any AI research project or any similar multi-disciplinary teamwork and perform as educators regarding the use of AI in medicine.

## The situation in Japan

S. Aoki, on behalf of the Japan Radiological Society (JRS), reported that the JRS is engaged in several actions in order to cope with, co-exist and master AI. He explained that some universities in Japan, such as the Kyoto Prefectural University of Medicine, have started daily case conferences thus facilitating communications between radiologists who work in different cities. Radiologists are currently at the centre of these conferences with other departments and at the centre of management and utilization of image information. S. Aoki also reported that the JRS is creating a nation-wide diagnostic imaging database called the Japan Medical Imaging Database (J-MID), which collects radiation exposure records, DICOM images and diagnostic imaging reports. In the J-MID project, the JRS is collaborating with the Japanese National Institute of Informatics to develop an artificial intelligence system for subarachnoid haemorrhage detection using datasets obtained from multiple institutions. S. Aoki also stated that the University of Tokyo is currently working on boosting the broad capabilities of radiologists by applying generative models such as *Glow* to generate images that emphasize deviations from normal distributions. Since this method does not depend on supervised learnings by annotating specific image findings by human radiologists, it is expected that is will reduce the annotation cost and will be able to emphasize any abnormal findings.

## The situation in the Asia-Oceania region

D. Varma, on behalf of the Asian Oceanian Society of Radiology (AOSR), stated that the work of radiologist is not only interpreting imaging findings but more importantly being integral to a clinical team deciding if an imaging is indicated and justified and if so, what is the most appropriate imaging that will progress patient care as well as timely communication of significant findings. He pointed out that radiologists are more and more visible and clinically orientated and that AI, unless it is moderated by a human being, in this context, the radiologist, will not be able to be harnessed in the manner that will be safe for the patient. Therefore, a radiologist that is not visible and that does not use AI will be the one that is replaced by AI. D. Varma also pointed out that mutual communication between clinicians and radiologists can never be replaced by AI and that skill-based examinations, such as ultrasound or interventional procedures, cannot be replaced by AI no matter how delicate the robot is. On the other hand, AI can enhance the safety and efficiency of a radiologist’s work, helping to pick up accidentally missed lesions, prioritise the examinations waiting for reporting and improve the quality of radiology reports. D. Varma concluded that AI would add value to radiologists in reducing errors and triage of workflow; however, if the “invisible” radiologists choose to be completely dependent on AI, they will be the most at risk of being replaced by it.

## The situation in Australia and New Zealand

L. Lawler, reporting on behalf of the Royal Australian and New Zealand College of Radiologists (RANZCR), stated that AI and machine learning technologies offer wide-ranging benefits to radiology and health care in general, but they will not replace radiologists in the foreseeable future as radiologists have a central and critical role in clinical decision-making and are becoming increasingly accessible to patients and referrers through multi-disciplinary care teams and advances in interventional radiology. L. Lawler explained that the adoption of AI presents many opportunities to support and enhance current workflows, allowing for a more efficient and accessible healthcare system that delivers improved outcomes for patients and that the RANZCR have been leaders in the adoption of AI in health care for the past 3 years. In August 2019, RANZCR became the first healthcare body in the world to develop and release *Ethical Principles for Artificial Intelligence in Medicine* [9] and complemented these with the subsequent release of *Standards of Practice for Clinical Radiology* [10] in September 2020. RANZCR is currently developing a position paper on the regulation of AI in medicine in Australia and New Zealand. L. Lawler pointed out that RANZCR believe that robust regulation of AI as a medical device is paramount in protecting patients and that adequate consideration must be given to the potential of an AI tool to cause harm. RANZCR’s other priorities are introducing profession-led implementation and workforce upskilling to support the effective implementation of AI into clinical practice. L. Lawler also stated that, if done well, AI could help deepen radiologists’ understanding of disease profiles and management of complex patients. Thinking beyond diagnosis and differential diagnoses, the potential of AI systems as prognostic and predictive tools is significant. L. Lawler concluded the report by stating that the key shift that AI presents for radiologists is the need to adapt and transform their role alongside the technology to ensure clinical oversight and guidance at every stage. The safe and ethical implementation of AI should ensure that it enables more efficient health care; however, it is not a suitable replacement for human-led clinical practice.

## The situation in Europe

A. Rockall reported on behalf of the European Society of Radiology (ESR). She explained that media attention in 2015 brought radiology into the spotlight and opened a wide conversation concerning the role of the radiologist in value-based patient care [11], an important topic that was also touched upon in the 2019 ESR paper *Value-Based Radiology: A New Era Begins* [12]. This in turn led to a communal reflection by those in radiology, as well as clinicians, patients and healthcare providers, on what radiologists offer in the widest sense, as explained in the ESR paper on the identity and role of the radiologist in 2020 [13] that was based on a survey carried out among ESR full radiologist members. A. Rockall pointed out that the recent pandemic has highlighted the need for humans in adapting to the rapid changes required in new and emerging diseases and that radiologists have been central to the recognition of patterns of disease, including the multi-organ findings and thrombotic events [14, 15]. Adapting imaging diagnostics and workflows is not something that could be replaced by AI. Many AI tools have been swiftly developed, but it is evident that none has been able to replace the complexity of the task faced by radiologists in adapting to the needs of the pandemic. A. Rockall emphasized that the expertise of radiologists is critical in shaping AI developments, initially by identifying pinch points in workflow, as well as diagnostic challenges that would be suitable use cases and that radiology training needs to incorporate the essentials of AI development and testing, as well safe and ethical use of AI tools. When it comes to workflow AI tools, A. Rockall states that these are more than welcome and only enhance the efficiency of radiologists. Concerning patient-facing roles, such as ultrasound and interventional procedures, A. Rockall states that these are not perceived to be under threat by AI. AI tools that support time-consuming tasks, such length or volume measurements or diagnostic tools, such as for pulmonary nodule detection, may improve efficiency. However, the radiologist provides the essential final assessment and remains accountable to patients, ensuring safe care. Commenting on the potential time savings caused by the AI, A. Rockall points out that, despite potential workforce shortages, it is important to advocate for increased face-to-face communication with patients and clinical teams. Radiologists, who contribute to multi-disciplinary meetings with colleagues, become central to team discussions of complex cases, integrating the clinical, histological and imaging information. In conclusion, A. Rockall stated that the advent of AI could propel radiology into a high visibility technology-enabled speciality and that departments that embrace AI may attract the brightest and the best trainees that wish to work in the exciting world of technology-enhanced medical imaging and diagnostics. Radiologists that are involved in planning and developing AI solutions, training AI-enabled radiologists of the future and implementing thoughtful and ethical AI into imaging diagnostics will ensure the very high visibility of radiology as a profession.

## Position of the International Society of Radiology (ISR)

R. A. Mendonça, on behalf of the International Society of Radiology (ISR), reported that the ISR collaborated in the *WHO Rapid Advice Guide on the Use of chest imaging in COVID-19* [16], which identified the study of the role of artificial intelligence in chest imaging in different settings as one of the research priorities. Pointing out the importance of visibility R. A. Mendonça also mentioned the joint WHO-ISR webinar on diagnostic imaging in sports medicine held in celebration of the International Day of Radiology (IDOR) 2019. The ISR is also in official relations with the IAEA and collaborates with the IAEA Radiation Protection of Patients Unit and the IAEA Nuclear Medicine and Diagnostic Imaging Section and regularly participates in IAEA Technical Meetings and related development of guidance documents and training material. Commenting on the role of the AI, R. A. Mendonça stated that the ISR sees AI as a means to support radiologists and consequently health systems in further enhancing the quality and affordability of imaging. The Lancet Oncology Commission on Medical Imaging and Nuclear Medicine, which counts the ISR among its supporting organisations, dedicates a section to the potential of advances in digital sciences and device engineering for improving cancer care in LMICs. In the ISR webinar on 'AI cannot replace a well-trained radiologist' held in March 2020, Prof. Y.-H. Chou concluded that well-trained radiologists are superior to mere AI, not least due to the aspect of human morality which they add. Finally, R. A. Mendonça concluded that the ISR attaches high importance to its collaboration with IAEA, WHO and other international stakeholders to increase the visibility of radiologists. The Covid-19 pandemic has highlighted the importance of imaging, and radiologists will have to continue raising awareness of their vital role in this and other communicable and non-communicable diseases. It will be essential to clarify the role of AI as an indispensable tool which radiologists must embrace, which will, however, benefit patients only if used appropriately by radiologists thanks to their expertise and ability to consider also ethical aspects.

## Conclusion

The future of radiology might be here sooner than anticipated. Despite initial apprehension many radiologists today believe that AI will optimize radiologists' workflows and develop new avenues for practicing radiology. Crucial element in that regard is going to be the increased visibility of radiologists which can only be achieved through the use of AI. In that sense only radiologists who embrace the possibilities of AI and are open to change will be able increase their visibility and establish themselves as an irreplaceable part of the medical teams of the future.

Radiologists around the world are experiencing fundamental shifts in their profession brought with the advent of AI. However, AI is creating new opportunities and is not only increasing the level of care that can be provided to the patients but is at the same allowing radiologists to redefine their role. Instead of making the radiologists obsolete AI will allow them to reposition themselves and increase their visibility by positioning them technology leaders in the inevitable change that will affect the whole of medical profession.

## Data Availability

All data generated or analysed during this study are included in this published article.

## Abbreviations

ACR: American College of Radiology
ACR: Colombian Association of Radiology
AI: Artificial intelligence
AOSR: Asian Oceanian Society of Radiology
CBR: Brazilian College of Radiology
CIR: Inter-American College of Radiology
CSR: Chinese Society of Radiology
DSI: ACR’s Data Science Institute
ECR: European Congress of Radiology
ESR: European Society of Radiology
ESRNM: Egyptian Society of Radiology and Nuclear Medicine
FDA: US Food and Drug Administration
FMRI: Mexican Federation of Radiology and Imaging
IRIA: Indian Radiological and Imaging Association
ISR: International Society of Radiology
J-MID: Japan Medical Imaging Database
JRS: Japan Radiological Society
KSR: Korean Society of Radiology
MOU: Memorandum of Understanding
PACS: Picture Archive and Communication System
RANZCR: Royal Australian and New Zealand College of Radiologists
RCRT: Royal College of Radiologists of Thailand
RICORD: RSNA International COVID-19 Open Radiology Database
RIS: Radiology Information System
RSE: Radiological Society of the Emirates
RSNA: Radiological Society of North America
RSSA: Radiological Society of Saudi Arabia
RST: Radiological Society of Thailand
SMRI: Mexican Society of Radiology and Imaging
SPR: Radiological and Diagnostic Imaging Society of São Paulo
TCELS: Thailand Centre of Excellence in Life Sciences
TCIA: The Cancer Imaging Archive
